# In dogs undergoing extrahepatic portosystemic shunt attenuation, does pretreatment with levetiracetam reduce postoperative seizure incidence?

**DOI:** 10.18849/ve.v7i3.581

**Published:** 2022-08-17

**Authors:** Connor Hawes+, Kali Lazzerini+

**Keywords:** CANINE, LEVETIRACETAM, PORTOSYSTEMIC SHUNT, SEIZURES, SHUNT ATTENUATION

## Abstract

PICO question

In dogs undergoing surgical attenuation of a congenital extrahepatic portosystemic shunt, does pretreatment with levetiracetam reduce the incidence of post attenuation seizures?

Clinical bottom line

Category of research question

Treatment

The number and type of study designs reviewed

Four papers were critically reviewed. All were retrospective cohort studies

Strength of evidence

Moderate

Outcomes reported

In one paper levetiracetam was found to reduce the risk of post-attenuation seizures. In the remaining three papers no difference was found between the frequency of post-attenuation seizures and the use of levetiracetam

Conclusion

That prophylactic levetiracetam is not indicated for the use of preventing post-attenuation seizures in dogs surgically treated for extrahepatic portosystemic shunts

How to apply this evidence in practice

The application of evidence into practice should take into account multiple factors, not limited to: individual clinical expertise, patient’s circumstances and owners’ values, country, location or clinic where you work, the individual case in front of you, the availability of therapies and resources.

Knowledge Summaries are a resource to help reinforce or inform decision making. They do not override the responsibility or judgement of the practitioner to do what is best for the animal in their care.

## Clinical scenario

A 6 month old Yorkshire Terrier diagnosed with a single congenital portosystemic shunt has surgical attenuation with an ameroid constrictor following medical management with lactulose, amoxicillin and a hydrolysed diet. The dog develops post-attenuation seizures 24 hours after surgery, despite no prior history of seizures. Would the administration of levetiracetam, an anti-epileptic drug, prior to attenuation reduce the risk of post-attenuation seizures?

## The evidence

Four retrospective cohort studies were found relevant to the PICO (Fryer et al., 2011; Mullins et al., 2018; Otomo et al., 2020; and Strickland et al., 2018). All studies compared the frequency of post-attenuation seizures in patients treated with or without prophylactic levetiracetam. Prior medical treatment, surgical technique, anaesthetic, frequency of preoperative neurological signs and levetiracetam protocols varied between and within studies, which all may have influenced seizure frequency. Fryer et al. (2011) was the only study to find a clinical benefit to prophylactic levetiracetam, the other three studies found no benefit (Mullins et al., 2018; Otomo et al., 2020; and Strickland et al., 2018). The frequency of post-attenuation seizures were low in all studies.Currently the evidence does not support the use of levetiracetam, although a prospective controlled study with standardised treatment protocol would be required.

## Summary of the evidence

**Otomo et al. (2020):** 

Population:	Dogs with single extrahepatic portosystemic shunts underwent gradual attenuation with a thin film band (TFB) or ameroid ring constrictor (ARC) at two institutes between 2004–2017. Criteria for exclusion: intrahepatic portosystemic shunt (IHPSS), multiple shunts.
Sample size:	123 dogs.
Intervention details:	85 dogs were treated with a TFB. 64/85 (75%) received prophylactic levetiracetam. 38 dogs were treated with an ARC. 15/38 (40%) received prophylactic levetiracetam. Surgical technique was based on surgeon’s preference. No standardised pre- or postoperative treatment. No information on the decision to give levetiracetam was reported.
Study design:	Retrospective cohort.
Outcome studied:	Short term <30 days and long term >6 months outcome were studied. Cases were categorised as successful or unsuccessful. Successful – no longer have clinical signs or require medication. Unsuccessful – still on medication, have clinical signs or died.
Main Findings (relevant to PICO question)	10/123 (8%) dogs had postoperative seizures at <30 days, 9/10 (90%) of which died. Levetiracetam was not shown to reduce the risk of seizures. 7/79 (9%) dogs treated with levetiracetam and 3/44 (7%) without levetiracetam had seizures p >0.99. There was no statistical difference in the number of dogs with postoperative seizures and the presence or absence of preoperative neurological signs p = 0.19, or surgical technique used p = 0.17.
Limitations:	Retrospective study. Only two institutions. Post-attenuation seizures were defined as occurring <30 days post operation and may include other causes of seizures. Non-randomised treatment groups – dogs thought to be higher risk of seizures may have been treated with levetiracetam. No record of levetiracetam protocol. No grading of severity of preoperative neurological signs. Non-standardised medical treatment or anaesthetic protocol.

**Mullins et al. (2018):** 

Population:	All dogs treated surgically, either with suture ligation, thin film banding (TFB), or an ameroid ring constrictor (ARC)I for a single extrahepatic portosystemic shunt at 10 different institutes between 2005–2017. Exclusions: intrahepatic portosystemic shunt (IHPSS), multiple shunts, 24 hour postoperative death not related to seizures, previous anti-epileptic drugs.
Sample size:	940 dogs.
Intervention details:	523 control dogs received no anti-seizure prophylaxis. Dogs were divided into two treatment groups LEV1 and LEV2. LEV1 consisted of 188 dogs which received levetiracetam at a dose of ≥15 mg/kg every 8 hours for ≥24 hours preoperatively or a 60 mg/kg intravenous loading dose peri-operatively, with continuation postoperatively at a dose of ≥15 mg/kg every 8 hours. LEV2 consisted of 229 dogs which received levetiracetam at a dose of <15 mg/kg every 8 hours, for <24 hours preoperatively, or continued at <15 mg/kg every 8 hours postoperatively. Treatment groups were non-randomised and decided by the clinician. Other treatments were not standardised.
Study design:	Retrospective cohort.
Outcome studied:	Focal or generalised seizures at less than or equal to 7 days postoperative, dogs which developed seizures at greater than 7 days were not classified as having post-attenuation seizures.
Main Findings (relevant to PICO question)	75/523 (8%) dogs had postoperative seizures, 35/523 (7%) control dogs, 21/188 (11%) LEV1 dogs, 19/229 (8%) LEV2 dogs. There was no statistical difference between the frequency of seizures and treatment group p = 0.14. There was no significant difference between signalment, treatment or previous seizure history and the presence of postoperative seizures. All postoperative seizure dogs were still on levetiracetam at the time of seizure.
Limitations:	Treatment groups were not randomised. Retrospective study. No grading of severity of preoperative neurological signs. Non-standardised medical treatment or anaesthetic protocol.

**Strickland et al. (2018):** 

Population:	All dogs that underwent surgical attenuation for a single intra or extrahepatic congenital portosystemic shunt at a single centre between 2000–2015.
Sample size:	253 dogs.
Intervention details:	148 dogs received partial attenuation, 105 complete attenuation. The decision to attenuate partially was based on perioperative portal pressure and visual assessment of the pancreas and intestinal tract. 12/148 (8%) dogs which received partial attenuation were with a cellophane band, and the remaining with suture ligation. 54 dogs received prophylactic levetiracetam at a dose of 20 mg/kg orally every 8 hours for a minimum of 24 hours preoperative and 5 days postoperative. Prophylactic levetiracetam was started in patients from 2012 onwards. 238 dogs received preoperative medical treatment.
Study design:	Retrospective cohort.
Outcome studied:	Survival to discharge. Presence of post-attenuation neurological signs (PANS) up to discharge graded on severity from 1–3. Changes to albumin osmolality, urea, glucose and electrolyte pre and postoperatively.
Main Findings (relevant to PICO question)	3/54 (6%) dogs receiving levetiracetam had seizures, 9/199 (5%) dogs not receiving levetiracetam had seizures. Risk of postoperative seizures was not associated with prophylactic levetiracetam use p = 0.75. Of the five dogs with seizures and did not survive to discharge, none received levetiracetam.
Limitations:	Non-randomised treatment groups. Retrospective data. Non-standardised treatment protocol. Dogs only received levetiracetam after 2012 and other factors may have also changed at this time influencing seizure frequency.

**Fryer et al. (2011):** 

Population:	All dogs which received an ameroid ring constrictor (ARC) for a single congenital extrahepatic portosystemic shunt at a single institution between 2003–2010. Exclusion criteria: previous treatment with anti-epileptic drugs.
Sample size:	126 dogs.
Intervention details:	All dogs received an ARC. 42 dogs received prophylactic levetiracetam at a median dose of 60 mg/kg/day for a median duration of 6.5 days preoperative to 33 days postoperative. All dogs that received levetiracetam were recruited after 2007. 84 dogs received no anti-epileptic drugs.
Study design:	Retrospective cohort.
Outcome studied:	Presence, number, type, timing and treatment response of post-attenuation seizures as recorded on hospital documents for a minimum of 48 hours.
Main Findings (relevant to PICO question)	Four dogs (3%) developed post-attenuation seizures, none of which received prophylactic levetiracetam. There was no significant difference in the frequency of preoperative neurological signs or seizures between the levetiracetam treated and non-treated dogs. Levetiracetam was found to significantly reduce the risk of seizures. Risk <1, p = 0.0002.
Limitations:	Retrospective study. Only a single institution. Inconsistent dose and duration of levetiracetam. No dogs received levetiracetam prior to 2007, and other changes to the management of cases may have influenced seizure frequency. No grading of severity of preoperative neurological signs. Low seizure frequency, low power to study. Preoperative medical management is not described.

## Appraisal, application and reflection

Following attenuation of a portosystemic shunt approximately 5–18% will experience post-attenuation seizures as a complication (Gommeren et al., 2010; Hardie et al., 1990; and Tisdall et al., 2000). These seizures typically occur within 72 hours of attenuation, are refractory to treatment and are associated with a high mortality (Gommeren et al., 2010). The pathophysiology of this condition is poorly understood and may be associated with a reduction in endogenous benzodiazepines, alongside postoperative metabolic events (Hardie et al., 1990; and Matushek et al., 1990), and may represent a number of aetiologies. Reported risk factors for post-attenuation seizures include: increased age (Hardie et al., 1990; Matushek et al., 1990; Strickland et al., 2018; and Tisdall et al., 2000), porto-azygos shunts (Tisdall et al., 2000), pre-existing signs of hepatic encephalopathy (Strickland et al., 2018), and increase serum osmolality (Strickland et al., 2018). Because of the lack of predictive factors or effective treatment there is a growing interest in developing preventative measures for post-attenuation seizures. One such treatment is the anti-epileptic drug levetiracetam, used for the treatment of status epilepticus, focal and generalised seizures, as well as not being contraindicated in hepatic dysfunction (Packer et al., 2015).

Fryer et al. (2011) was the first paper to explore the use of prophylactic levetiracetam, and the only study suggesting a benefit. No patients treated with levetiracetam had post-attenuation seizures, whereas 4/84 4.8% of patients not treated did. Despite the promising results the further three papers reviewed showed no benefit to levetiracetam (Mullins et al., 2018; Otomo et al., 2020; and Strickland et al., 2018). Mullins et al. (2018) and Strickland et al. (2018) also had substantially larger samples sizes and seizure frequencies compared to Fryer et al. (2011). Based on this it can be concluded that prophylactic levetiracetam does not reduce the risk of post-attenuation seizures. Strickland et al. (2018) did suggest that the use of prophylactic levetiracetam did reduce the mortality associated with post-attenuation seizures, although frequency of seizures and number of patients on levetiracetam were low.

The major limitation in all studies were other factors potentially contributing to post-attenuation seizures, and being able to determine if the seizures were secondary to other factors. No study was consistent in the use of anaesthetic protocol, surgical technique, and use of preoperative medication, all of which may contribute to seizure frequency. The use of levetiracetam was also not consistent, with varied protocols, which may alter its efficacy. Lastly the presence of preoperative neurological signs and seizures varied largely between studies ranging from 64/123 (52%) (Otomo et al., 2020), 85/125 (68%) (Fryer et al., 2011), 61/75 (81%) (Mullins et al., 2018), and 253/253 (100%) (Strickland et al., 2018). Strickland et al. (2018) was the only study to try and grade preoperative neurological signs, although did not appear to consider grade in their analysis. The presence of preoperative hepatic encephalopathy is considered a risk factor for post-attenuation seizures (Strickland et al., 2018), and severity of preoperative neurological signs may be an important source of bias not considered in all of these studies.

In conclusion the evidence does not support the use of prophylactic levetiracetam in reducing post-attenuation seizures, levetiracetam may be useful in reducing mortality associated with this condition although further studies would be required to conclude this.

## Methodology Section

**Search Strategy:** 

Databases searched and dates covered:	CAB Abstracts on the OVID interface, 1973–2022 Week 01 PubMed on the NCBI interface. 1920–January 2022
Search strategy:	CAB Abstracts: (dog or dogs or canine or canines).mp. or exp dogs/ (congenital or primary).mp. (portosystemic or portasystemic or porto-systemic or porta- systemic or shunt* or PSS or cPSS or cEHPSS or extrahepatic or extra-hepatic).mp. (levetiracetam or antiseizure or antiepileptic or anticonvulsant or anti-seizure or anti-epileptic or anti-convulsant).mp. PubMed: #1 dog or canine #2 congenital or primary #3 portosystemic or portasystemic or porto-systemic or porta-systemic or shunt or PSS or cPSS or cEHPSS or extrahepatic or extra-hepatic #4 levetiracetam or antiseizure or antiepileptic or anticonvulsant or anti-seizure or anti-epileptic or anti-convulsant #5 #1 and #2 and #3 and #4
Dates searches performed:	12 Jan 2022

**Exclusion / Inclusion Criteria:** 

Exclusion:	Not related to PICO. Review paper. Book chapter. Foreign language.
Inclusion:	Research papers including the use of levetiracetam in the prevention of post-attenuation seizures even if not the primary aim.

**Search Outcome:** 

Database	Number of results	Excluded – Not related to PICO	Total relevant papers
CAB Abstracts	7	3	4
PubMed	10	6	4
Total relevant papers when duplicates removed			4

## Conflict of interest

The authors declare no conflicts of interest.
